# Retraction: NF-κB activation by *Helicobacter pylori *requires Akt-mediated phosphorylation of p65

**DOI:** 10.1186/1471-2180-11-128

**Published:** 2011-06-02

**Authors:** Eriko Takeshima, Koh Tomimori, Hirochika Kawakami, Chie Ishikawa, Shigeki Sawada, Mariko Tomita, Masachika Senba, Fukunori Kinjo, Hitomi Mimuro, Chihiro Sasakawa, Jiro Fujita, Naoki Mori

**Affiliations:** 1 Division of Molecular Virology and Oncology, Graduate School of Medicine, University of the Ryukyus, 207 Uehara, Nishihara, Okinawa 903-0215, Japan; 2 Division of Control and Prevention of Infectious Diseases, Graduate School of Medicine, University of the Ryukyus, 207 Uehara, Nishihara, Okinawa 903-0215, Japan; 3 Division of Child Health and Welfare, Faculty of Medicine, University of the Ryukyus, 207 Uehara, Nishihara, Okinawa 903-0215, Japan; 4 The Japanese Society for the Promotion of Science (JSPS), Japan; 5 Division of Oral and Maxillofacial Functional Rehabilitation, Faculty of Medicine, University of the Ryukyus, 207 Uehara, Nishihara, Okinawa 903-0215, Japan; 6 Department of Pathology, Institute of Tropical Medicine, Nagasaki University, 1-12-4 Sakamoto, Nagasaki 852-8523, Japan; 7 Department of Endoscopy, University Hospital, University of the Ryukyus, 207 Uehara, Nishihara, Okinawa 903-0215, Japan; 8 Department of Microbiology and Immunology, Institute of Medical Science, The University of Tokyo, 4-6-1 Shirokanedai, Minato-ku, Tokyo 108-8639, Japan

## Abstract

Article retracted

## Retraction

After lengthy investigation by the editors, the original article [1] has been retracted because of inappropriate duplication of images from previously published articles. The last author, Naoki Mori takes full responsibility and apologizes for any inconvenience caused.
